# Plastid DNA sequences and oospore characters of some European taxa of *Tolypella* section *Tolypella* (Characeae) identify five clusters, including one new cryptic *Tolypella* taxon from Sardinia, but they do not coincide with current morphological descriptions

**DOI:** 10.3389/fpls.2023.1096181

**Published:** 2023-03-01

**Authors:** Anja Holzhausen, Petra Nowak, Andreas Ballot, Ralf Becker, Jasmina Gebert, Thomas Gregor, Kenneth G. Karol, Elisabeth Lambert, William Pérez, Uwe Raabe, Susanne C. Schneider, Nick Stewart, Klaus van de Weyer, Volker Wilde, Hendrik Schubert

**Affiliations:** ^1^ Institute of Biological Sciences, Aquatic Ecology, University Rostock, Rostock, Germany; ^2^ Department of Biology, Plant Cell Biology, University Marburg, Marburg, Germany; ^3^ Norwegian Institute for Water Research, Oslo, Norway; ^4^ Independent Researcher, Oldenburg, Germany; ^5^ Senckenberg Forschungsinstitut und Naturmuseum, Botanik und Molekulare Evolutionsforschung, Frankfurt am Main, Germany; ^6^ Program for Molecular Systematics Studies, The New York Botanical Garden, Bronx, NY, United States; ^7^ Département de Biologie Environnement, Faculté des Sciences, Université Catholique de l’Ouest, Angers, France; ^8^ Independent Researcher, Marl, Germany; ^9^ Independent Researcher, Glastonbury, United Kingdom; ^10^ lanaplan GbR, Nettetal, Germany; ^11^ Paläontologie und Historische Geologie, Paläobotanik, Senkenberg Forschungsinstitut und Naturmuseum, Frankfurt am Main, Germany

**Keywords:** charophytes, genetic diversity, oospore morphology, phylogeny, *Tolypella*, taxonomic concepts

## Abstract

In Europe, the genus *Tolypella* (Characeae) comprises four to eight *Tolypella* taxa in sections *Rothia* and *Tolypella* that have been distinguished by vegetative morphology and gametangial characters such as antheridial size and oospore wall ornamentation. However, morphological differentiation is difficult in some cases due to overlapping and variable vegetative features, which in many cases are difficult to observe clearly. To clarify the taxonomic status of the five European taxa of *Tolypella* in section *Tolypella*, sequence data of the plastid genes *atp*B, *rbc*L and *psb*C for *Tolypella glomerata* (Desv.) Leonh., *Tolypella hispanica* Allen, *Tolypella nidifica* (O.F. Müll.) A. Braun, *Tolypella normaniana* (Nordst.) Nordst. and *Tolypella salina* Cor. were combined with data on oospore morphology, including oospore wall ornamentation. Gene sequence data identified five distinct clusters, but they were not consistent with the morphologically identified five taxa. *T. glomerata* consisted of some of the samples morphologically identified as *T. glomerata* and seven samples of *T. normaniana*, while the remaining *T. glomerata* samples clustered with specimens of unclear affiliation (*Tolypella* sp.). We identified two clusters of *T. hispanica* within the European material: cluster *T. hispanica* I consisted of samples from various locations, whereas the second cluster (*T. hispanica* II) consisted of samples of *T. hispanica* from Sardinia Island. The remaining cluster consisted of all the specimens that had been determined as *T. salina* or *T. nidifica* in addition to two specimens of *T. normaniana*. Oospore morphology was most clearly distinguishable for *T. glomerata*. Oospore characteristics for all other taxa were not as informative but showed some geographical and/or environmentally influenced differences, especially for *T. nidifica* and *T. salina*. Our results suggest the need to further check the different taxonomy of *Tolypella* sect. *Tolypella* in which specimens normally identified as *T. glomerata* might be two different taxa, *T. glomerata* and an unidentified taxon; *T. nidifica* and *T. salina* are not separate taxa; *T. normaniana* is a diminutive variant of two different *Tolypella* taxa; and *T. hispanica* comprises two different taxa, one from the Mediterranean island Sardinia.

## 1 Introduction

Charophytes, extant and fossil members of the order Charales plus the members of the extinct orders Sycidiales and Moellerinales (Schneider et al., 2015) are algae with a complex morphology, which are closely related to modern land plants (Nishiyama et al., 2018). Taxa delineation of charophytes is commonly based on morphological traits of the plant thallus, and accurate identification of charophytes is important for understanding their diversity and for documenting changes in distribution. Charophyte identification is, however, hampered because of morphological plasticity influenced by abiotic factors. This specifically applies to the genus *Tolypella* A. Braun, where morphological characters are in some cases difficult to use because of (1) their small size and fragility, which often makes characters hard to observe; (2) phenotypic plasticity due to environmental influences such as water level and salinity (Lambert et al., 2013; Mouronval et al., 2015); and (3) their short vegetative cycle that (a) often impede the use of characters derived from mature oospores (Wood, 1965) and (b) lead to fewer taxa collections due to their main development period being within a short time period that is easily missed. Some Characeae, particularly in the genus *Tolypella*, are ephemeral and seem to be rare. Most authors agree to split *Tolypella* into two sections, *Tolypella* and *Rothia*, differentiated mainly by the shape of end cells (obtuse for *Tolypella* and acute for *Rothia*, Krause, 1997) as well as habitat traits (Mouronval et al., 2015). Former *Tolypella* “unranked” *Obtusifolia*, described by Allen (1883) became a synonym of *Tolypella* sect. *Tolypella* by the choice of *Tolypella nidifica* as type of *Tolypella* by Wood (1965). This section includes taxa with evanescent and obtuse end cells, undivided sterile branchlets and a separated basal impression (Sawa and Frame, 1974). Eight taxa of *Tolypella* have been described from Europe (Krause, 1997): five taxa are included in section *Tolypella* and include *Tolypella glomerata* (Desv.) Leonh., *Tolypella hispanica* Allen, *Tolypella nidifica* (O.F. Müll.) A. Braun, *Tolypella normaniana* (Nordst.) Nordst., and *Tolypella salina* Cor. Taxa of the section *Rothia* are not considered in this study. There is no agreement about the taxonomic status of these taxa among different authors. For example, *T. nidifica* and *T. salina* were treated as distinct taxa by Krause (1997) or Mouronval et al. (2015) based on oospore features including ornamentation patterns, while Corillion (1960) described *T. salina* as new taxon based on morphological and cytological criteria.

Of all the taxa in section *Tolypella*, only *T. hispanica* can be unambiguously differentiated, because they are dioecious, while all other *Tolypella* taxa are monoecious. Identification of the remaining four taxa has been mainly based on vegetative morphological traits, including oospore characteristics (Krause, 1997) and ecological features (Wood, 1965). In order to aid identification of the genus *Tolypella*, the additional use of oospore characteristics (e.g. length, number of striae and membrane ornamentation) has been suggested as occasionally useful (e.g. Pérez et al., 2015). In addition, DNA barcoding, i. e. the use of short regions of DNA to identify taxa by assigning individuals to known taxa through comparison of their barcodes with a reference library, has become a popular means to improve identification (Mccourt et al., 1999; Sheth and Thaker, 2017). Moreover, DNA barcoding permits the identification of morphologically similar but genetically different (‘cryptic’) taxa (Bickford et al., 2007; Struck et al., 2018), a common phenomenon for algae (Díaz-Tapia et al., 2018). Pérez et al. (2016) used the genes, *atp*B, *rbc*L and *psb*C successfully for discrimination within the genus *Tolypella* in North America. Therefore, the same three plastid genes were also used in this study to investigate the diversity of section *Tolypella*.

The aim of this study is to gain new insights into European *Tolypella* taxa by means of oospore characters combined with genetic data. For this, specimens of *T. glomerata*, *T. hispanica*, *T. normaniana*, *T. salina* and *T. nidifica* were examined, with the latter two included in such an attempt for the first time.

## 2 Materials and methods

### 2.1 Vegetative morphology and gametangial characters

Fresh plant material was morphologically determined by the respective collector (**Tables S1**, **S2**) based on descriptions by various authors (Wood and Imahori, 1965; Corillion, 1975; Krause, 1997; Cirujano et al., 2008; Lambert et al., 2013; Pérez et al., 2016; Van De Weyer and Schmidt, 2018). According to those, all used *T. hispanica* were clearly identified by their dioecious character, whereas *T. glomerata*, *T. salina* and *T. nidifica* were first determined by antheridial sizes, habitat occurrences and oospore ornamentation.

Individuals that featured vegetative and antheridial characters of two taxa, e.g*., T. nidifica* and *T. salina*, were determined as *T.* sp.

### 2.2 Material

Specimens identified as *T. glomerata*, *T. hispanica*, *T. nidifica*, *T. normaniana* and *T. salina* by means of vegetative characters (mostly antheridia sizes) were obtained from herbarium collections and from field collections by the authors for a total of 157 specimens. The collections span the period between 1871 and 2020 from locations in nine European countries (Austria, France, Germany, Great Britain, Greece, Ireland, Italy, the Netherlands, Norway, Portugal, Sweden) and from Chile in South America (**Table S1**). In addition, 40 specimens (Denmark, Italy, Germany, Great Britain, Greece, France, Norway, Portugal, Sweden) could not be unambiguously assigned morphologically to any recognized taxon and are referred to as *Tolypella* sp. throughout the manuscript.

Specimens of *T. nidifica* and *T. glomerata* from deep water sites were collected by diving. In shallow waters, samples were gathered by snorkeling or wading. Some specimens of *T. salina* (France) and *T. glomerata* (Germany) originated from germination experiments under laboratory conditions (e.g., Holzhausen, 2016; Holzhausen et al., 2017). For all fresh material, oospores were harvested after release from cultured material in order to confidently assess oospore maturity. In addition, oospores of *T. nidifica* from Austria and Germany were collected from sediment samples. Herbarium specimens were sampled from collections deposited in the Herbarium Rostochiense (ROST), the New York Botanical Garden Sterre Herbarium (NY), UiO Vascular Plants Herbarium, Natural History Museum, University of Oslo (O) and private herbaria of the collectors (**Table S1**).

### 2.3 Genetic analyses

Dried plant material was obtained from a total of 193 individuals initially identified as *T. glomerata* (60 specimens), *T. hispanica* (15 specimens), *T. nidifica* (51 specimens), *T. salina* (50 specimens), *T. normaniana* (9 specimens), and 13 morphologically ambiguous *Tolypella* sp. Genetic data for the *atp*B, *psb*C and *rbc*L plastid genes presented in this study were obtained by three different working groups: A) the University of Rostock, B) the New York Botanical Garden and C) the Norwegian Institute for Water Research by the following methods.

Method A) Genomic DNA was extracted using the DNeasy Plant Mini Kit (Qiagen, Hilden, Germany), following the manufacturer’s protocol. Amplification of the plastid genes *rbc*L, *psb*C, and *atp*B was performed with 10 PCR cycles with one minute each of annealing at 94°C, extension at 55°C, and denaturation at 72°C, followed by one minute each for denaturation (94°C), annealing (52°C), and polymerisation (72°C) in 25 cycles. The amplified DNA was purified using the Biometra-innuPrep Gel ExtractionKit (Analytik Jena, Jena, Germany) according to the manufacturer’s instructions. Samples were sequenced using a 3130×L GeneticAnalyzer (Applied Biosystems, NY, USA) with sequencing primers identical to the primers that were used for PCR reactions (**Table S3**). Obtained sequences were checked visually and aligned using BioEdit v.7.0.5.2 (Hall, 1999).

Method B) Genomic DNA was extracted using the Nucleon Phytopure DNA extraction kit (GE Healthcare Gio-Sciences, Pittsburgh, PA, USA, Pérez et al., 2014). The *atpB, psbC* and *rbcL* genes were amplified by a nested PCR reaction using either a PTC-200 DNAEngine^®^ Thermal Cycler (Bio-Rad, Hercules, CA, USA) or a Mastercycler^®^ pro S (Eppendorf AG, Hamburg, Germany). Initial PCR amplicons were generated through the following cycling program: initial denaturation at 95 ˚C for 2 minutes; 35 cycles of 95 ˚C for 15 seconds; 48 ˚C for 15 seconds and 72 ˚C for 30 seconds; and followed by a final extension at 72 ˚C for five minutes. The resulting PCR product were used in a second round of PCR amplification to generate internal sequences using the same cycling program with the exception that the cycling was reduced to 30 cycles and the final extension time reduced to 30 seconds. Products from both PCR sets were sequenced at the University of Washington Genome Center (Seattle, WA, USA).

Method C) Genomic DNA from *Tolypella* material was isolated after (Schneider et al., 2016). PCR for the *rbc*l, *atp*B, and *psb*C genes was performed on a Bio-Rad CFX96 Real-Time PCR Detection System (Bio-Rad Laboratories, Oslo, Norway) using the iProof High-Fidelity PCR Kit (Bio-Rad Laboratories, Oslo, Norway). The following cycling protocol was used for all three genes: one cycle of 5 min at 94°C, and then 35 cycles each consisting of 10 s at 94°C, 20 s at 62°C, and 20 s at 72°C, followed by a final elongation step of 72°C for 5 min. PCR products were visualized by 1.5% agarose gel electrophoresis with GelRed staining (GelRed^®^ Nucleic Acid Gel Stain, Biotium, Fremont, USA) and UV illumination. Amplification of the *rbc*L, *atp*B and *psb*C gene region was conducted using the primers listed in **Table S3**. In some cases, a nested PCR was conducted using the former PCR product as template and a second primer pair for a further PCR amplification. For sequencing the same primers and if necessary, intermediate primers were used (**Table S3**). Sequences were analyzed and aligned using Seqassem (version 04/2008) and Align (version 03/2007) MS Windows-based manual sequence alignment editor (SequentiX – DigitalDNA Processing, Klein Raden Germany) to obtain DNA sequence alignments, which were then corrected manually. For each PCR product, both strands were sequenced on an ABI 3730 Avant genetic analyzer using the BigDye terminator V.3.1 cycle sequencing kit (Biosystems, Applied Biosystems, Thermo Fisher Scientific Oslo, Norway) according to the manufacturer’s instructions.

Complete sequences of all three plastid genes could not be generated for every sample analyzed due to the differing qualities of the specimens (age, storing conditions, drying conditions after collection, etc.). Therefore, four different datasets were used for the phylogenetic analyses in order to obtain as much information as possible for all specimens. The first dataset included the three plastid gene sequences from 88 individuals, whereas three additional data sets were compiled for each plastid gene separately; the *atp*B dataset with a total of 1034 positions for 94 samples, the *psb*C dataset with a total of 1104 positions for 125 samples, and the *rbc*L dataset with a total of 1265 positions for 189 samples. As outgroup, which is defined as closely related taxon or group outside of the taxon investigated, two different sequences of *T. porteri* were used. In order to estimate evolutionary divergence, pair-wise uncorrected p-distances and the number of substitutions were conducted using MEGA version7 (Kumar et al., 2016). To uncover phylogenetic relationships, Bayesian inference (BI) and maximum likelihood (ML) trees were constructed, with evolutionary substitution models evaluated in MEGA v.7. The method selected the same best-fitting evolutionary model (GTR+G+I) for each of the four datasets. The ML algorithm was conducted in MEGA v.7 with 1000 bootstrap replicates. BI trees were performed with MrBayes 3.2.6 (e.g. Ronquist and Huelsenbeck, 2003) with a random starting tree and two independent runs of one cold and three heated chains, each using default parameters. Each analysis was run for 2 million generations with trees sampled every 1000 generations and the first 25% generations discarded as burn-in.

Due to small genetic distances among some taxa, intraspecific data often produce a variety of possible trees when using conventional tree building methods. In such cases, the relationship among taxa is best expressed by a network that is able to show alternative potential phylogenetic relationships within a single figure (Bandelt et al., 1999). Furthermore, networks allow the identification and illustration of ancestral alleles whereas phylogenetic trees treat all sequences as terminal taxa (Posada and Crandall, 2002). For that reason, Median-Joining (MJ) network analyses were performed using the PopART software v1.7 (Leigh et al., 2015).

### 2.4 Oospore analyses

The terminology of oospore characters in this study is based on Soulié-Märsche and García (2015). Descriptions of membrane ornamentation follow those of Frame (1977) and Urbaniak et al. (2012). Altogether, 712 mature oospores were harvested from herbarium specimens, fresh plant material and sediment samples. Oospores were collected from 12 specimens of *T. glomerata*, 19 of *T. nidifica*, 18 of *T. salina* and 10 of *T.* sp. The individual numbers of oospores examined and the appropriate pre-treatments are given in **Table S2**. Oospores were stored in well plates for eventual re-examination and are part of the Rostocker oospore database (Holzhausen et al., 2015).

For stereomicroscopic analysis, oospores were photographed in lateral, apical, and basal views with a mounted camera. Qualitative oospore characteristics that were examined included colour, shape and membrane ornamentation. To differentiate among the various brown hues of oospores, colour terms used in this study are clay brown, fawn brown, nut brown, chestnut brown, dark brown wine red and black brown (RAL COLOUR SYSTEM). Quantitative characteristics included: number of striae, expression of striae (prominence of striae), angle of striae with respect to the longitudinal axis, oospore length and width, fossa width (average of 4 fossae), and length of the outer lines of the pentagonal basal impression. Length measurements were calculated using ImageJ 1.50i. The ISI (isopolarity index; 100*(length/width)) was also calculated.

Scanning electron microscope (SEM) analyses of oospores were performed at the Senckenberg Forschungsinstitut und Naturmuseum Frankfurt. Prior to SEM observations, few oospores were pre-cleaned (HoAc 5%) and all were dried by lyophilisation. Dry specimens were later sputter-coated with gold. SEM images of the surface of oospores and fossa walls (magnification 200-2500X) were produced with a JEOL JSM-6490 LV in high-vacuum mode by using secondary electrons and routinely applying an acceleration voltage of 20kV.

Oospore characters were tested for normality using the Shapiro-Wilk Test. Pairwise tests were performed for different levels of analyses (taxon, country, region, type of location and plants) by the Kruskal-Wallis Test (SPSS). P ≤ 0.05 was used as statistical significance for oospore analyses.

To identify (a) the correlation between oospore characteristics and regionality and (b) parameter combinations that might provide reliable discrimination, combined analyses of all quantitative and qualitative oospore features as well as their ratios, with the exception of the membrane ornamentation, were performed by nonmetric multivariate techniques using the Primer7 software package (Clarke and Gorley, 2015). Principal component analysis (PCA) was based on standardised, square root transformed data and Euclidean distance matrices. Multiplot-Analysis based on non-treated raw data.

## 3 Results

### 3.1 Genetic analyses

**Figure 1:**
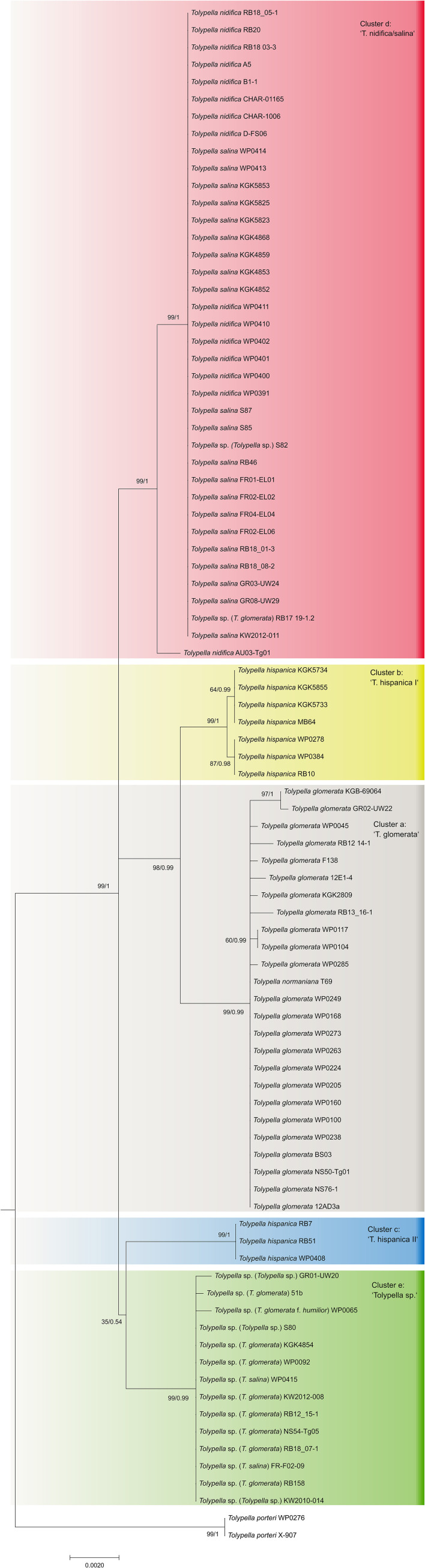
Maximum likelihood tree of genus *Tolypella* based on concatenated *atp*B, *psb*C, and *rbc*L sequence data. Phylogeny of Characeae based on combined *atp*B, *psb*C, and *rbc*L sequence data. Maximum likelihood tree with bootstrap values and posterior probabilities above branches (≥ 50%).

**Figure 2:**
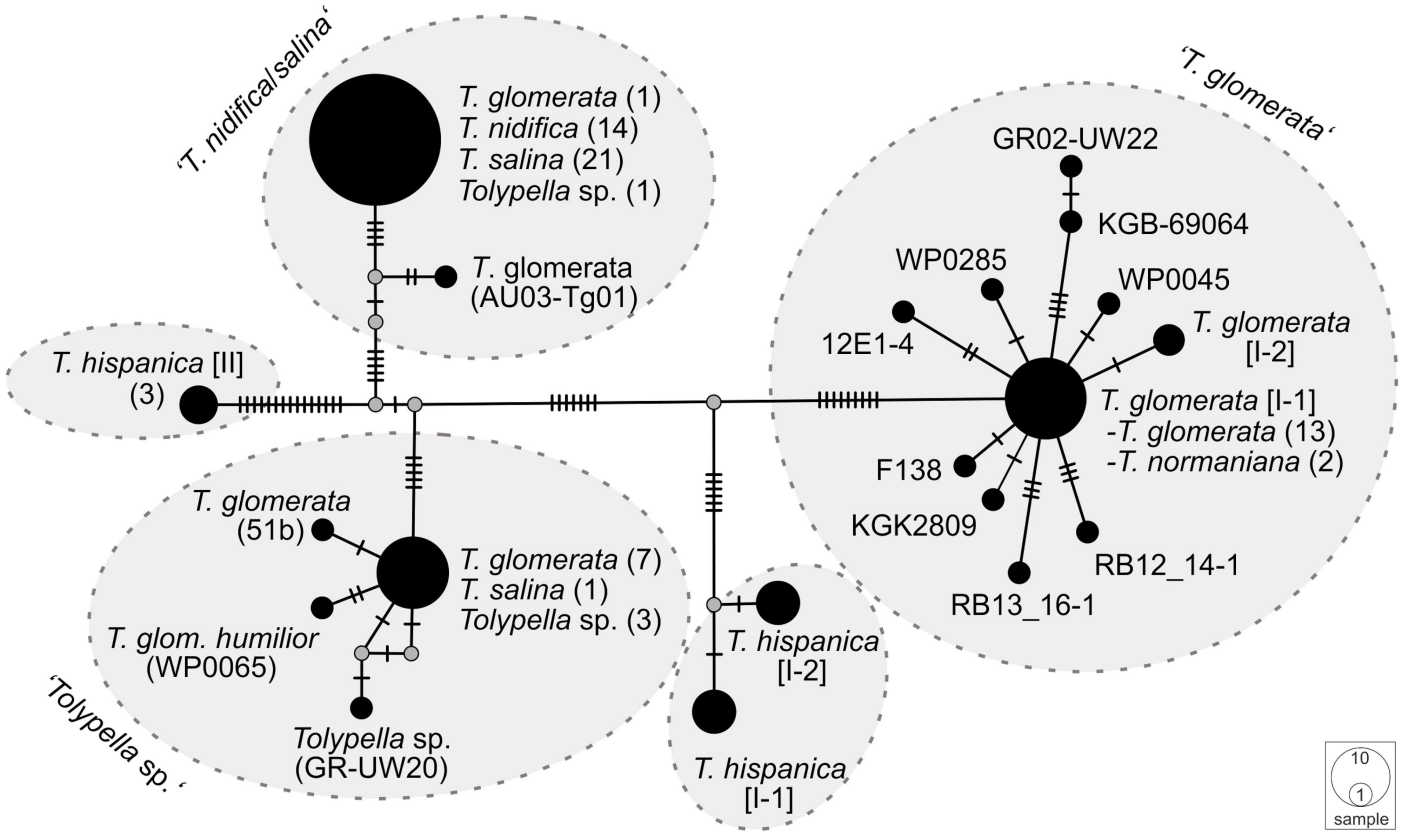
A Median Joining network of concatenated *atp*B, *psb*C, and *rbc*L sequences of sec. *Tolypella*. Circles represent haplotypes, with the size being proportional to their relative frequencies. The smallest circle corresponds to a single haplotype copy. A small black line at branches indicates one inferred mutational step. The small grey dot is a median vector and represents a possible extant unsampled haplotype or an extinct ancestral haplotype.

The phylogenetic analyses of the plastid gene sequences in each dataset recovered the 196 *Tolypella* individuals into five general clades that were denoted as ‘*T. glomerata*’, ‘*T. nidifica*/*salina’*, ‘*Tolypella* sp.’, and two distinct ‘*T. hispanica*’ clades (‘*T. hispanica* I’ and ‘*T. hispanica* II’; **Figure 1**; **Table S4**). Supporting values for each cluster were given in the sections below. The gene sequence similarities of *Tolypella* individuals within each group were generally over 99.8% (**Table 1**). However, support for their phylogenetic placements were unresolved or weakly to moderately supported in the single gene analyses. Phylogenetic resolution and support were greatest in the three-gene analyses. The results of the network analyses were comparable to the phylogenetic trees, with the same clusters recovered in both approaches (**Figures 1**, **2**; **Table S1**; **Figures S1**–**S3**). Results for the ML tree and the MJ network of concatenated gene sequences are shown in **Figures 1**, **2**. Complete trees and networks for single gene analyses are shown in the supplement (**Figures S1**–**S3**). The labels used to identify genetic groups correspond to those in **Table S1**.

**Table 1:** Estimates of evolutionary divergence based on concatenated dataset over sequence pairs between (black) and within (blue) main groups.

	*T. glomerata*	*Tolypella* sp.	*T. nidifica*/*salina*	*T. hispanica* I	*T. hispanica* II
*T. glomerata*	0.12% | 3.85	27.73	26.77	18.00	31.92
*Tolypella* sp.	0.85%	0.06% | 2.00	19.40	24.00	23.40
*T. nidifica*/*salina*	0.82%	0.59%	0.01% | 0.38	23.50	23.97
*T. hispanica* I	0.55%	0.73%	0.72%	0.06% | 2.00	30.00
*T. hispanica* II	1.01%	0.73%	0.72%	0.92%	0.00% | 0.00

Shown are the number of base differences (lower left) and the pairwise uncorrected p-distances (upper right) per sequence from averaging over all sequence pairs between and within groups.

*‘T. glomerata’*

A first group comprising mostly *T. glomerata* contained 49 individuals that represented *T. glomerata* (39 specimens), *T. normaniana* (7 specimens), and *T.* sp. (1 specimen). Specimens originated from nine European countries (France, Germany, Great Britain, Greece, Ireland, Italy, Netherlands, Norway, Sweden), and from the United States, Canada, and Chile. The analysis of concatenated sequences of ‘*T. glomerata*’ identified a difference of ≥ 18 nucleotides with respect to other clusters of sect. *Tolypella* (**Table 1**). With an average of about 3.85 bp substitutions, the genetic variability within the group was relatively high compared to the intragroup variability shown by the other taxa. However, differences in their sequence data were not regionally correlated; European and North American specimens showed identical sequences. In contrast, 8 substitutions were observed between samples collected in Greece (GR02-UW22) and Italy (RB13_16-1 and RB12_14-1).

*‘T. nidifica/salina’*

A second cluster (labelled ‘*T. nidifica*/*salina*’) consisted of 110 individuals which have traditionally been assigned mainly to the taxa *T. nidifica* (44 specimens) and *T. salina* (50 specimens). Additionally, two *T. normaniana*, and 14 morphologically ambiguous *T.* sp. were found in this cluster (**Table S4**). They were collected in Austria, France, Germany, Greece, Italy, Norway and Sweden. Interestingly, the specimen originally determined as *T. glomerata* f. *littorea* from France (KGK4867) and two of nine sequenced *T. normaniana* (T70/T71) clustered within *T. nidifica/salina*. Sequence data for each of the *atpB*, *psbC* and *rbcL* genes could not be obtained for every specimen in this cluster. r*bc*L sequence data was obtained from 188 specimens, whereas sequences for *atpB* and *psbC* were obtained from 38 specimens (**Table S4**). Overall, however, there was little genetic variation within the ‘*T. nidifica*/*salina*’ cluster when comparing each of the gene sequences. Depending on the dataset, between 95.2 and 97.6% of the analysed specimens had identical sequences. Minor genetic differences were observed in this group; two *T.* sp. collected from Italy (*rbc*L, **Figure S1**), and a *T. normaniana* from Norway (*psb*C, **Figure S3**) differed by a single nucleotide substitution each. Regional differences were not reflected in the sequence data with identical haplotypes throughout Europe. Consistent nucleotide differences were found only among two *T.* sp. collected in Austria (AU03-Tg01, AU03-Tg03).

*‘Tolypella sp.’*

A third cluster (labelled ‘*Tolypella* sp.’) consisted of 21 individuals which have been classified as morphologically ambiguous specimens due to the presence of vegetative characters of more than one *Tolypella* taxon mentioned above. The specimens were partly originally determined as *T. glomerata* f. *humilor.* They were collected in Denmark, France, Germany, Great Britain, Greece, Italy, Norway, Portugal, and Sweden. ‘*Tolypella* sp.’ revealed unique sequence data and differed from ‘*T. nidifica*/*salina*’ and ‘*T. glomerata*’ by averaging 19.4 and 27.9 bp substitutions respectively (**Table 1**). Nucleotide differences within ‘*Tolypella* sp.’ ranged from 0 to 4 bp substitutions for concatenated sequences (mean 0.06%, **Table 1**; **Figure 2**).

*‘T. hispanica’*

Two clusters labelled as ‘*T. hispanica* I’ and ‘*T. hispanica* II’ consisted of ten and five individuals, respectively, which have traditionally been assigned to the dioecious *T. hispanica*. ‘*T. hispanica* I’ included ten individuals collected in France, Greece, Italy, and Algeria. The samples collected in France had two unique nucleotide substitutions for the combined sequences (0.06%, **Table 1**). ‘*T. hispanica* I’ formed a strongly supported clade together with ‘*T. glomerata*’ (**Figure 1**). ‘*T. hispanica* II’ contained five individuals from four field collections in Italy, Sardinia that shared identical *rbc*L sequences whereas three individuals had identical sequences for all three genes (**Table 1**). In the ML analysis, however, ‘*T. hispanica* I’ was sister to ‘*Tolypella* sp.’ in a weakly supported relationship (**Figure 1**).

### 3.2 Oospore analyses

Differences in quantitative and qualitative oospore characters were considered with respect to taxa determined by either vegetative morphology or genetically determined cluster. The results of oospores grouped in taxa determined by vegetative morphology are summarized in **Table 2**.

**Table 2:** Oospore characteristics of *T. glomerata*, *T. nidifica/salina* and the morphologically unconclusive *T.* sp.

parameter	category	*T. glomerata*	*T. nidifica/salina*	*T.* sp.
colour (%)	clay brown	–	2.0	–
	fawn brown	97.4	–	8.4
	nut brown	–	0.2	–
	chestnut brown	1.7	32.6	7.4
	dark brown wine red	0.9	48.3	78.9
	black brown	–	16.9	5.3
shape (%)	terete	100	42.5	60.0
	globose	–	48.3	31.6
	ellipsoid	–	9.3	5.3
	peanut	–	–	3.2
striae (µm)		5 - 8 ( ± 0.9)	4 - 8 ( ± 0.7)	5 - 8 ( ± 0.7)
length (µm)		252.2 - 487.4 ( ± 49.3 - 50.4)	194.3 - 457.5 ( ± 45.1 - 45.3)	266.7 - 472.0 ( ± 39.5)
width (µm)		197.6 - 351.2 (± 34.1)	149.9 - 404.7 ( ± 40.5)	188.3 - 366.9 ( ± 27.9)
ISI		110 - 160 ( ± 10)	100 - 190 ( ± 10)	80 - 220 ( ± 20)
fossa (µm)		31.0 - 57.9 ( ± 6.3)	27.2 - 69.7 ( ± 6.5)	35.0 - 62.9 ( ± 6.0)
basis (µm)		29.0 - 61.8 ( ± 4,9 - 5.1)	27.1 - 89.9 ( ± 8.3 - 8.5)	30.5 - 61.4 ( ± 6.0)
angle (°)		62.3 - 87.9 ( ± 6.0)	51.3 - 89.2 ( ± 7.1)	56.3 - 86.9 ( ± 7.2)
expression of striae (µm)		0 - 17.9 ( ± 4.4)	0 - 17.7 ( ± 2.8)	3.0 - 15.1 ( ± 2.9)

Ranges of quantitative oospore features are given as min – max (± standard deviation).

*T. glomerata*

**Figure 3:**
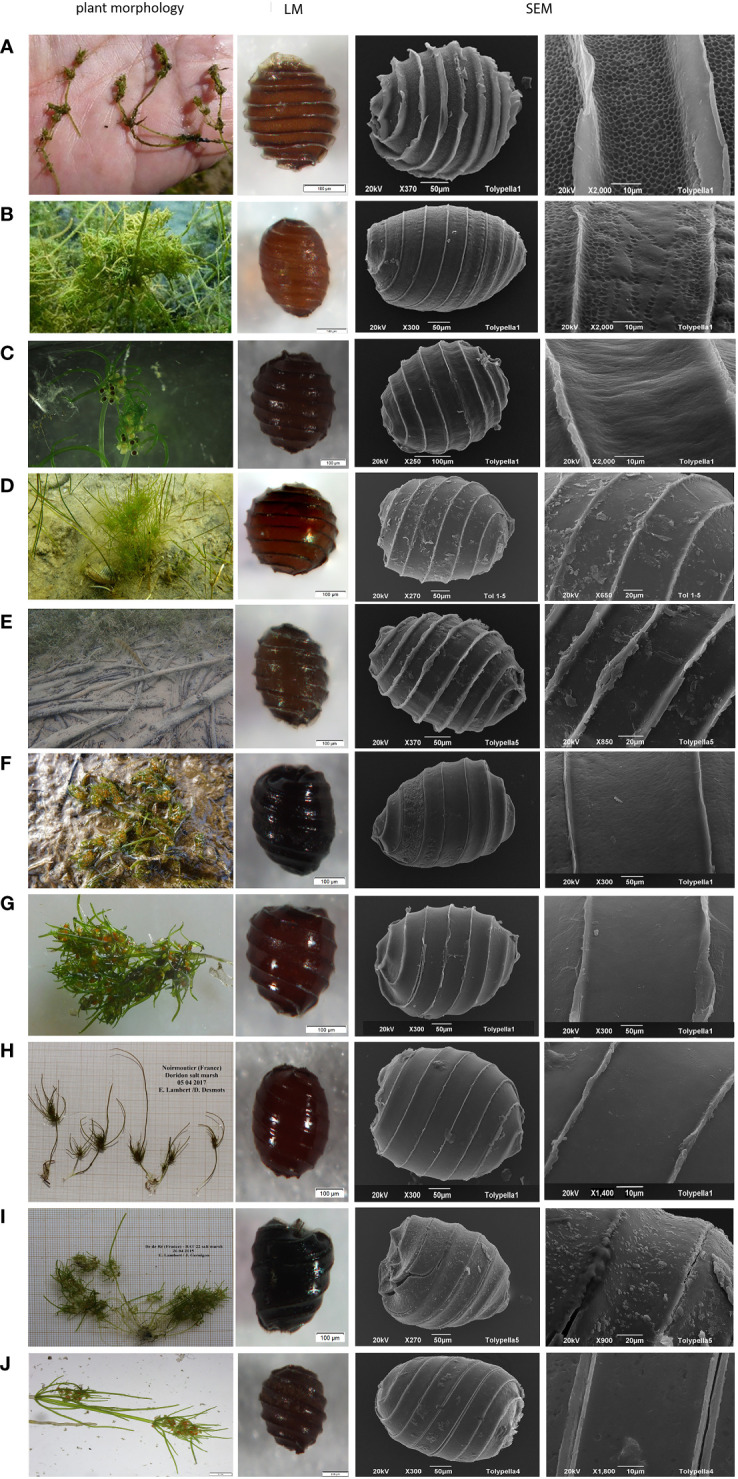
Habitus, LM and SEM of ‘*T. glomerata’*, ‘*T. nidifica’*, ‘*T. salina’* and ‘*T. sp*’. 3A - Habitus, LM and SEM of ‘*T. glomerata’* and ‘*T. nidifica’.* **(A)**-’*T. glomerata*’ RB13_16 (Italy, Cabras) with fully reticulate ornamentation pattern, **(B)**-’*T. glomerata*’ BS-Tol (Germany, Borkener See) with partially reticulate ornamentation of oospores. **(C)**-’*T. nidifica*’ TN3-1 (Germany, Fehmarn), **(D)**-’*T. nidifica*’ Tol04 (Germany, Lehmkenhafen), **(E)**-’*T. nidifica*’ Tol7 (Austria, Apetlon Badesee), 3B - Habitus, LM and SEM of ‘*T. nidifica’*, ‘*T. salina’* and ‘*T. sp*’. **(F)**-’*T. nidifica*’ RB18_12 (Italy, Pittulongu). **(G)**-’*T. salina*’ RB18-01 (Italy, Pittulongu), **(H)**-’*T. salina*’ FR-EL/Sal1-07 (France, Île de Noirmoutiers), **(I)**-’*T.salina*’ FR-TS 687-01 (France, Île de Ré). **(J)**-’*T.* sp.’ FR-F02 (France, Kermadec).

Oospores analysed in this study were usually fawn brown (97.4%), occasionally chestnut brown (1.7%) or dark brown wine red (0.9%), with an elongated rounded shape with 7-8 striae. The expression of striae is flat to prominent (0.0–17.9µm). Oospores showed lengths of 252.2 to 487.4 µm ( ± 49.8-50µm), widths from 197.6 to 351.2 µm ( ± 33.5-34.1µm), a mean fossa width of 31.0 to 57.9 µm, mean lengths of the outer basal impression from 29.0 to 61.8 µm and an ISI of 110–160. All oospores exhibited a reticulate ornamentation in varying expression and size (**Figure 3**; **Table S2**).

*T. nidifica/salina*

Oospores of *T. nidifica/salina* were mainly dark brown wine red (48.3%), chestnut brown (32.6%) or black brown (16.7%) with a terete or broad rounded/globose shape and a flattened base. Oospores showed (4-)5-7(- 8) striae that were flat and prominent (0.0–17.7 µm), oospore lengths of 194.3–457.5 µm ( ± 45.2µm), oospore widths of 149.9–404.7 µm ( ± 40.4µm), mean fossa width of 27.2–69.7 µm and an outer mean basal impression length of 27.1 to 89.9 µm. The calculated ISIs ranges between 100 and 190. Ornamentation patterns of *T. nidifica/salina* were highly variable, from smooth to smooth with some pustules or with fine linear structures (**Figure 3**; **Table S2**).

*Tolypella sp.*

The oospores of the morphologically ambiguous specimens were dark brown wine red (78.9%), occasionally fawn brown (8.4%), chestnut brown (7.4%) or black brown (5.3%) with a broad range of shape variations (ellipsoid, elongate rounded, broad rounded/globose, or peanut- shaped). Oospores showed (5) 6–7 (-8) striae with widths between 3.0–11.2 µm, oospore lengths of 266.7–472.0 µm ( ± 38.7µm) and widths between 195.6–346.7 µm ( ± 27.9 µm). Fossae ranged from 37.2 µm to 62.2 µm and outer basal impression lengths from 33.1 up to 57.9 µm. Calculated ISIs ranges from 80 to 190 (-220). The membrane of *T.* sp. Oospores was smooth to smooth with some pustules or with fine linear structures (**Figure 3**; **Table S2**).

**Figure 4:**
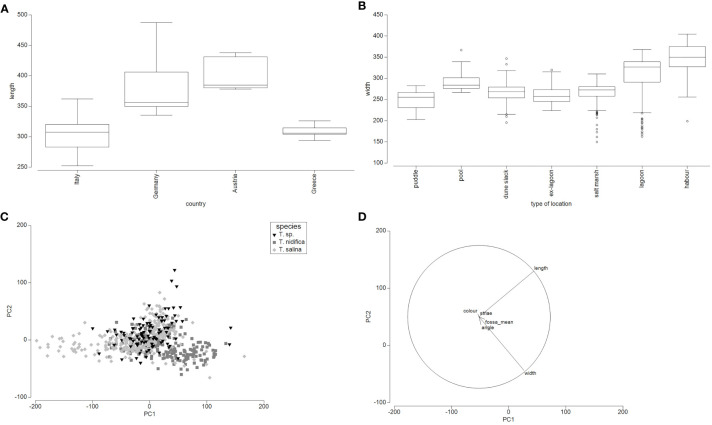
Analyses of oospore features. **(A)**- Box-Plot of oospore length in µm of *T. glomerata* depending on the country. **(B)**- Box-Plot of oospore widths (µm) of locally separated *T. glomerata* populations. **(C)** - PCA of vegetative determined *T. nidifica*, *T. salina* and *T.* sp. Oospores depending on the country. Included were 565 oospores from Germany, France and Italy. **(D)** – Oopsore characters of the PCA of vegetative determined *T. nidifica*, *T. salina* and *T.* sp.

The analysis of taxon-related oospore characteristics shows that variations, especially with regard to the features colour, shape and length exist within each taxon (**Figure 3**; **Tables 2**, **S2**). Especially for *T. glomerata*, large discrepancies between oospores along a geographical gradient and between populations could be observed (**Figure 4**). The PCA shows that the two axes explain 64.8% of the cumulative variation of oospores (eigenvalue 1 = 6.72, eigenvalue 2 = 3; **Table S5**). The first component is determined by the characters length and width and the ratios of length/angle and width/angle, whereas the second component is defined by the striae and width/fossa ratio. Depending on the level of analysis, significant intraspecific differences between countries, regions, type of locations and plants can be detected. Oospores from Germany (length: 335µm – 487µm, width: 255µm – 351µm) and Austria (length: 378µm – 438µm, width: 241µm – 313µm) were significantly larger and wider than those from Greece (length: 294µm – 326µm, width: 197µm – 221µm; p ≤.001) and Italy (length: 252µm – 362µm, width: 203-µm – 274µm; p ≤.001). No significant differences could be detected between oospores from Germany and Austria or between those from Italy and Greece.

Given the vegetative determination in *T. nidifica, T. salina* and *T.* sp., significant differences could be obtained for the oospore characters shape (p ≤.006), length (p ≤.005), fossa (p ≤.005) and width (p ≤.005) between *T. nidifica* and *T. salina*. *Tolypella* sp. could be separated from *T. nidifica* by the width, fossa (p ≤.006) or shape (p ≤.017) whereas *T.* sp. could be differentiated by oospore length (p ≤.005) and shape (p ≤.006) from *T. salina* although overlapping areas exist. However, these differences can only be obtained by means of statistical tests. Multiplot Analysis (**Figure S4**) on the other hand show the overlapping areas, which were partly caused by single plants or individual oospores. Due to the lack of statistical power, they should be seen only as trend.

Interestingly, depending on the type of location, oospores of permanent water bodies (lagoons, harbours and lakes) and pools are wider than oospores from temporary water bodies (**Figure 4**).

## 4 Discussion

The results of genetic analyses for European *T. glomerata* and *T. salina/nidifica* specimens can be confirmed only partly by oospore analyses. However, few examples showed that identified genetic differences could be confirmed by oospore features, especially wall ornamentation pattern. The results are only partially consistent with the current phenetic taxonomic concept (Groves and Bullock-Webster, 1920; Corillion, 1975; Krause, 1997; Cirujano et al., 2008; Mouronval et al., 2015). Both analyses can confirm differences of unambiguous *Tolypella* specimens to *T. glomerata* and *T. nidifica/salina*. Based on genetic analyses they are located within the *‘Tolypella* sp.’ Cluster, whereas oospore analyses revealed significant differences in e.g., length and width. In contrast to the sequence data, significant differences could be detected between oospore lengths and widths of *T. nidifica* and *T. salina*. But these are mainly caused by local separations. However, Italian *T. nidifica* did not differ from French *T. salina* or Italian/French *T.* sp. This is in strong accordance with the results of sequence data. Differences in oospore ornamentation patterns were not reflected by sequence data of plastid genes (**Table S2**).

*Tolypella hispanica* is the only dioecious taxon in the section *Tolypella* and, by including sexuality as a taxonomically relevant parameter at species level, easily distinguished from all other European taxa. Several studies demonstrated that sex separation occurred independently in various groups of the Characeae (Proctor, 1980; Meiers et al., 1999; Pérez et al., 2016). Phylogenetic data of this study revealed that this taxon consists of two well-separated lineages, representing two cryptic taxa. One lineage (‘*T. hispanica* I’), in agreement with the results of Pérez et al. (2016), was related to *T. glomerata*. The second lineage (‘*T. hispanica* II’), identified for the first time in this study, was located very distant from ‘*T. hispanica* I’ and nearer to the cluster of ‘*Tolypella* sp.’ and ‘*T. nidifica/salina*’. The results of our analyses revealed the existence of two cryptic taxa, being united within the recent taxon *T. hispanica*. The existence of cryptic taxa is very common for about half of all marine eukaryotic organisms such as Rhodophyta (Payo et al., 2013), Chlorophyta (Cimino and Delwiche, 2002; Irisarri et al., 2021) or Phaeophyceae (Poong et al., 2013). In order to get a robust description of morphological characters for discrimination between the two dioecious taxa, detailed morphological and molecular analysis of a larger number of samples, including type specimens, are needed to resolve their taxonomic status. The herbarium material of *T. hispanica* did not exhibit fully mature oospores which could be included in this study. The use of unmature material is not reliable for such studies due to developmental differences of colour or ornamentation pattern, as it was shown for *Nitella* taxa by Casanova (1991).

Although wetland conservation is extremely important especially for Mediterranean islands such as Sardinia, Charophytes are not included in Sardinian conservation programmes so far. Becker (2019) highlighted the presence of 26 different charophyte taxa in Sardinia with respect to their habitat preference. Moreover, he suggested four different action plans for (I) Characeae of lagoons, temporary brackish pools, salt marshes and estuaries, (II) *Nitella* of temporary freshwater ponds and estuaries, (III) *Chara connivens* in temporary ponds and lakes and (IV) *Chara* of running waters in calcareous regions and water reservoirs to counteract the loss of taxa and habitats, including the new cryptic taxon belonging to *T. hispanica* II lineage.

Based on genetic analyses, we were unable to verify the rank of the morphologically determined *T. normaniana* (Langangen, 1994). Seven of nine specimens clustered within the *T. glomerata* cluster. One of the specimens from Nordland was sampled in 1870 and is genetically identified as dwarfed variant of *T. glomerata*. From the same region, a *T. normaniana* collected in 2005 (T69) is also genetically identified as *T. glomerata*. This indicates that *T. glomerata*, although not described from Norway yet, has occurred in this northern locality since at least 150 years (Langangen, 2021). Unfortunately, the available herbarium specimens do not have mature oospores, so that determination of wall ornamentation was not possible. Urbaniak et al. (2012) describes two types of ornamentation pattern with transitional forms, smooth and pitted, and concluded a strong relationship between *T. nidifica* and *T. normaniana*. In further studies, dwarf forms that morphological resemble *T. normaniana* should be analysed for oospore wall ornamentation, in order to assign to either *T. glomerata* or *T. nidifica/salina*.

For *T. glomerata*, a broad morphological variability within European specimens, comparable to the results published by Pérez et al. (2014) for North American specimens, was observed in this study. The shape of the whorls ranged from very compressed with short or long sterile branchlets to whorls which rather appear looser with long sterile branchlets (spike-like). This broad morphological variability is also reflected by oospore characters, exhibiting large regional differences (**Figure 3**). On the other hand, only small differences among gene sequences of European *T. glomerata* and those from North and South America could be detected (**Figure 2**). The results of this study show that even with a broader sampling range the ‘*T. glomerata*’ cluster remains stable. For North American specimens, Pérez et al. (2015) found a reticulate oospore ornamentation for *T. glomerata*, the most useful character for the distinction between *T. porteri* and *T. glomerata*. Antheridia size, on the other hand, seems not to be a suitable character for discrimination between *T. glomerata* and *T. nidifica*/*salina*. For example, eight individuals should be identified as *T. glomerata* using antherida sizes but were genetically determined as *T. nidifica*/*salina*. Although for *T*. *glomerata* smaller antheridia sizes in diameter (220–450µm) (Corillion, 1960; Krause, 1997) are reported than for *T*. *salina* (450–625µm (–1000µm) (Corillion, 1960; Krause, 1997; Cirujano et al., 2008; Lambert et al., 2013) and *T*. *nidifica* (450–550µm; (Krause, 1997; Urbaniak, 2003). This character is often used for discrimination between *T*. *glomerata* and *T*. *nidifica* in the field, but seems to be influenced by environmental conditions as reflected by a North-South gradient, resulting in a broad and overlapping size range for these taxa. Moreover, gametangial studies on *C. hispida* and *C. aspera* have shown that antheridia sizes depend on the whorl position (Calero and Rodrigo, 2022). Clear and unambiguous distinction between *T. salina* and *T. nidifica* could not be achieved by this study neither by means of genetic data nor by oospore morphology and ornamentation. The comparison of French and Italian *T. salina* (Lambert et al., 2013; Becker, 2019) with those of the Iberian Peninsula (Cirujano et al., 2008; Cirujano Bracamonte et al., 2013) depicts a large morphological variability, probably caused by environmental conditions. A correlation between habitat salinity and phenotypic plasticity/fructification has already been published for several halophytic charophytes (e.g., Winter and Kirst, 1991; Bonis et al., 1993).

Both *T. salina* phenotypes, (1) smaller plants with fewer and shorter fertile branchlets (Cirujano et al., 2008) and (2) bigger ones with a higher number and longer fertile branchlets and internodes (Lambert et al., 2013), have been identified in this study for France as well as Italy (**Figure 3**). Those interannual morphological variability is caused by environmental variability and well known for Characeae.

A similar large morphological variability appeared within *T. nidifica*. Specimens with very compact and compressed whorls, long branchlets and long internodes, as well as specimens with less compact and compressed whorls and shorter branchlets were observed (**Figure 3**). The same applies for oospore morphometry, also exhibiting large variability without being reflected by genetic differences of the standard marker genes investigated here.

Morphometric oospore characters exhibited site-specific and location-specific differences, but did not allow for discrimination between the two taxa. As for vegetative characters, the reason for this observed large variability might be habitat conditions such as (soil) salinity. Oospores from puddles could be clearly differentiated from higher saline locations such as salt marshes, lagoons or harbours. The Italian sites exhibited a salinity between 1.2 and 21.6g/L (Becker, 2019), the salinities of the French salt marshes ranged from 2.2 to up to 250g/L (Lambert et al., 2013). Large seasonal changes can be observed over the year and are mainly caused by drought and re-wetting of temporal ponds or puddles. In contrast, the salinity of the German location Lehmkenhafen (2020) shows lesser fluctuation with a salinity around 12.

*T. salina* was described by Corillion (1960) who distinguishes it from *T. nidifica* as the lower number of striae of the oospore (mostly 6 vs. 8) and smaller oospores (length 273–366 µm vs. 400–475 µm; width 258–312 µm vs. 350–450 µm). These differences could not be corroborated by us (**Table 2**).

Until now, besides the number of chromosomes (50 for *T. salina* and 20-42 for *T. nidifica*, (Corillion, 1960; Guerlesquin, 1967), the membrane ornamentation was found in this study as the most reliable character, although no clear distinction is possible. Whereas the oospores of *T. salina* showed a smooth ornamentation, excepting a few specimens with only few pustules, which is only partially in accordance with different authors (Corillion, 1960; Urbaniak et al., 2012), the membrane of *T. nidifica* exhibited in most cases pustules or linear structures, while only few specimens revealed smooth oospores. However, the number of oospores available for examination was rather low and, moreover, this result partly contradicts existing literature. Nordstedt (1889) described the oospore membrane of *T. nidifica* as smooth, Wood (1965); Ray et al. (2001) and Urbaniak et al. (2012) found a pit-like ornamentation for *T. nidifica* which ‘varied among populations’ and Corillion (1975) described both expressions. The results of this study also showed transitions between both ornamentation types which should be investigated in more detail as well as in correlation to the maturity status of oospores which was shown by Casanova (1991) for *Nitella* oospores.

In addition to overlapping morphological plant features, determinations may be hampered by the existence of intermediary forms between *T. glomerata* and *T. nidifica* as described for the French population from Herault (Hy, 1913; Corillion, 1957). So as for the vegetative characters, habitat-specific effects on oospore ornamentation needs to be investigated in more detail by physiological experiments before a definite conclusion about the reliability of ornamentation pattern for delineation can be made. However, a distinct genetic entity, until now represented just by one specimen, was detected. This specimen originated from a brackish lake near Apetlon in Austria/Burgenland (AU03-Tg01) and is the first record of *T. nidifica*/*salina* for Austria and should be investigated in more detail.

Consequently, a final conclusion about the taxonomic status of *T. nidifica*/*salina* cannot be made irrespective of the observed differences in ornamentation pattern. With respect to lacking genetic differences, Pérez et al. (2016) have shown that analyses based on ribosomal gene sequences support chloroplast data but are not reliable for discrimination between uncertain taxa.

Nevertheless, both analyses could be considered as appropriate, and imply possibilities for further investigations of the status of these European taxa such as analyses of geographically isolated *Tolypella* populations on the basis of Simple Sequence repeats as microsatellite studies have shown for the genus *Chara* (Schaible et al., 2009; Schaible et al., 2011; Noedoost et al., 2015). High-throughput sequencing techniques or multi-omic approaches including proteomics could be carried out to examine smallest genetic differences between populations as shown for *Nitellopsis obtusa* (Sleith and Karol, 2021).

## 5 Conclusions

This study showed that the combination of oospore morphology and sequence data are only partially consistent. Sequence data confirmed the taxonomic status of *T. glomerata* and *T. hispanica.* Besides this, a second dioecious *T. hispanica* lineage can be found.

Moreover, although *T. nidifica* and *T. salina* could not be separated by sequence data (`*T. nidifica/salina´)* and transitions in oospore ornamentation exist, this study reveals significant differences in oospore length and widths that are mainly caused by local differences. These results indicate that environmental factors affect oospore morphology. The rank of *T. normaniana* could not be confirmed by genetic results. Those individuals clustered within `*T*. *glomerata*´and `*T*. *nidifica*/*salina*´. Furthermore, the sequence data revealed a new genetic entity, currently named as *T.* sp. A final decision about the taxonomic status of *T*. *nidifica/salina* and *T*. sp. could not be done on the basis of these results. Nevertheless, all analyses could be considered useful, and imply possibilities for further investigations of the status of these European taxa.

## Data availability statement

The datasets presented in this study can be found in online repositories. The names of the repository/repositories and accession number(s) can be found in the article/**Supplementary material**.

## Author contributions

Conceptualization - AH; PN methodology – AH, AB, SS, TG, PN, KK, WP and VW; analysis/investigation: AH, AB, SS, TG, PN, KK, WP, VW, EL, UR, RB, JG and KvdW; resources – RB, EL, UR, NS, KvdW, and HS; original draft preparation - AH; review and editing – all authors, visualization - AH and PN. All authors contributed to the article and approved the submitted version.

## Supplementary material

Supplementary Figure 1 Phylogeny of Characeae based on *rbc*L sequence data. **(A)** Median Joining network of *rbc*L sequences of *Tolypella*. **(B)** Maximum likelihood tree of genus *Tolypella* based on *rbc*L sequence data with bootstrap values and posterior probabilities above branches (≥ 50%).

Supplementary Figure 2 Phylogeny of Characeae based on *atp*B sequence data. **(A)** Median Joining network of *atp*B sequences of *Tolypella*. **(B)** Maximum likelihood tree of genus *Tolypella* based on *atp*B sequence data with bootstrap values and posterior probabilities above branches (≥ 50%).

Supplementary Figure 3 Phylogeny of Characeae based on *psb*C sequence data. **(A)** Median Joining network of *psb*C sequences of *Tolypella*. **(B)** Maximum likelihood tree of genus *Tolypella* based on *psb*C sequence data with bootstrap values and posterior probabilities above branches (≥ 50%).

Supplementary Figure 4 Multiplot Analysis of *T. glomerata*, *T. nidifica*, *T. salina* and *T.* sp. Qualitative parameter are measured in µm.

Supplementary Table 1 List of the 195 *Tolypella* samples used for oospores and genetic analyses in the present study. The columns “oospore ornamentation” and “Genetics” columns indicate the results of oospore ornamental analysis as well as the DNA sequencing results. Detailed information is given in **Table S2** (oospore analyses) and **S4** (genetic analyses). The last columns show the accession numbers, with n.n. in case the genetic marker was not recovered. (Tglo=*T. glomerata*, Tni=*T. nidifica*, Tsal=*T. salina*, Tn/s=*T. nidifica*/*salina*, Thi=*T. hispanica*, Tsp=*T.* sp.). A - Nowak and Schubert, 2019, B -Pérez et al., 2014, C - Mccourt et al., 1999, D - Pérez et al., 2016; storage: 1 - Herbarium University Rostock, 2 New York Botanical Garden Steere herbarium, 3 - Vascular Plant Herbarium, National History Museum Oslo, 4 - Herbarium Vienna, 5 - Friener Herbarium, Butler University, 6 - United States National Herbarium) (Excel Data Sheet).

Supplementary Table 2 List of oospores used for SEM and REM analyses including oospore morphology and ornamentation and the irrespective genetic cluster. Kind of material: F - fresh plant material, H - herbarium specimen, S - sediment sample, G - germination experiment (Excel Data Sheet).

Supplementary Table 3 Oligonucleotide primers used in this study. “Method” refers to the method used for DNA-sequencing described in genetic analysis subsection in Materials and Methods (Excel Data Sheet).

Supplementary Table 4 List of 195 *Tolypella* specimens used for genetic analyses in the present study. The first column indicates the genetic identification of the sample, while the second column contains the morphological determination according to the plant characteristics. “Method” refers to the method used for DNA-sequencing described in genetic analysis subsection in Materials and Methods. The last four colums display the genetic cluster observed by analysing single gene sequence data and a combined data set. Cluster names correspond to **Figure S1** (*rbc*L), **Figure S2** (*atp*B) and **Figure S3** (*psb*C), and , (*atp*B + *rbc*L + *psb*C).

Supplementary Table 5 List of eigenvalues and eigenvectors resulted from the principal component analysis of all *Tolypella* samples including absolute oospore values and their ratios.
