# Supplementary figures and images for: Plastid DNA sequences and oospore characters of some European taxa of Tolypella section Tolypella (Characeae) identify five clusters, including one new cryptic Tolypella taxon from Sardinia, but they do not coincide with current morphological descriptions
Source: Front Plant Sci. 2023 Mar 1;14:1096181. doi: 10.3389/fpls.2023.1096181 (PMC10014841; doi:10.3389/fpls.2023.1096181)

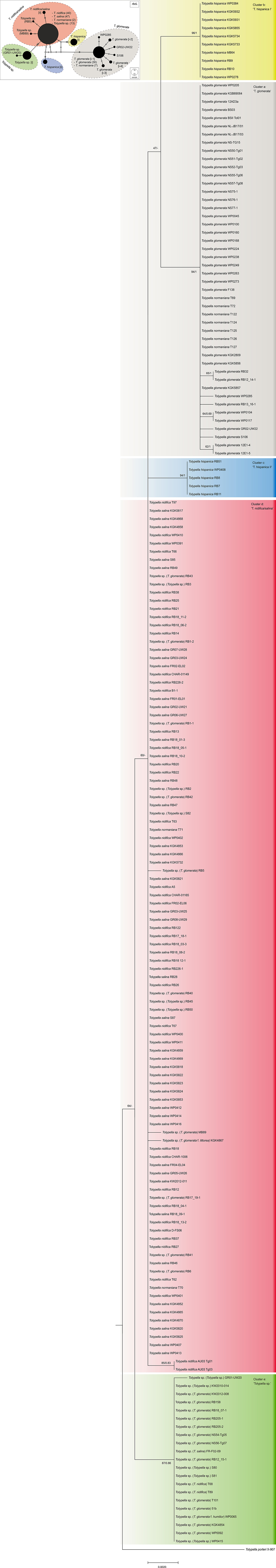

Supplement: Supplementary Figure 1 — Phylogeny of Characeae based on rbcL sequence data. (A) Median Joining network of rbcL sequences of Tolypella. (B) Maximum likelihood tree of genus Tolypella based on rbcL sequence data with bootstrap values and posterior probabilities above branches (≥ 50%). [file Image_1.jpeg]

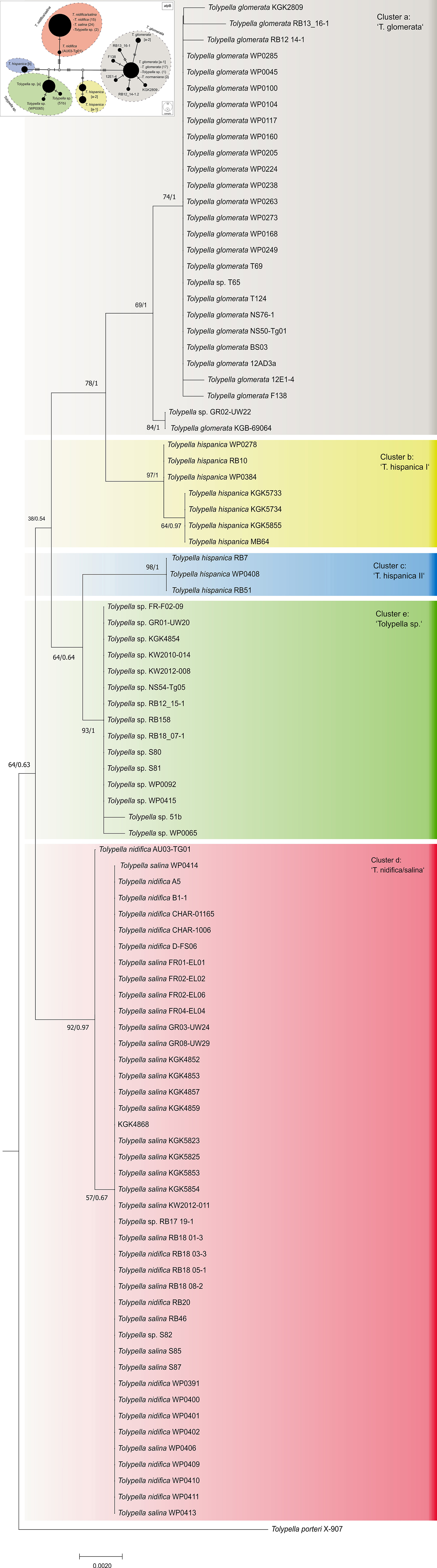

Supplement: Supplementary Figure 2 — Phylogeny of Characeae based on atpB sequence data. (A) Median Joining network of atpB sequences of Tolypella. (B) Maximum likelihood tree of genus Tolypella based on atpB sequence data with bootstrap values and posterior probabilities above branches (≥ 50%). [file Image_2.jpeg]

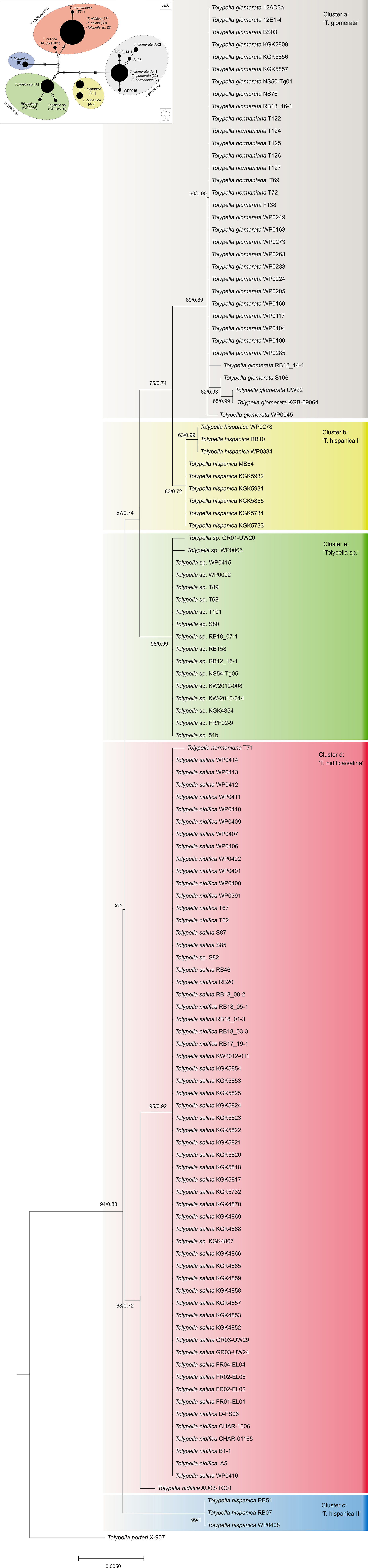

Supplement: Supplementary Figure 3 — Phylogeny of Characeae based on psbC sequence data. (A) Median Joining network of psbC sequences of Tolypella. (B) Maximum likelihood tree of genus Tolypella based on psbC sequence data with bootstrap values and posterior probabilities above branches (≥ 50%). [file Image_3.jpeg]

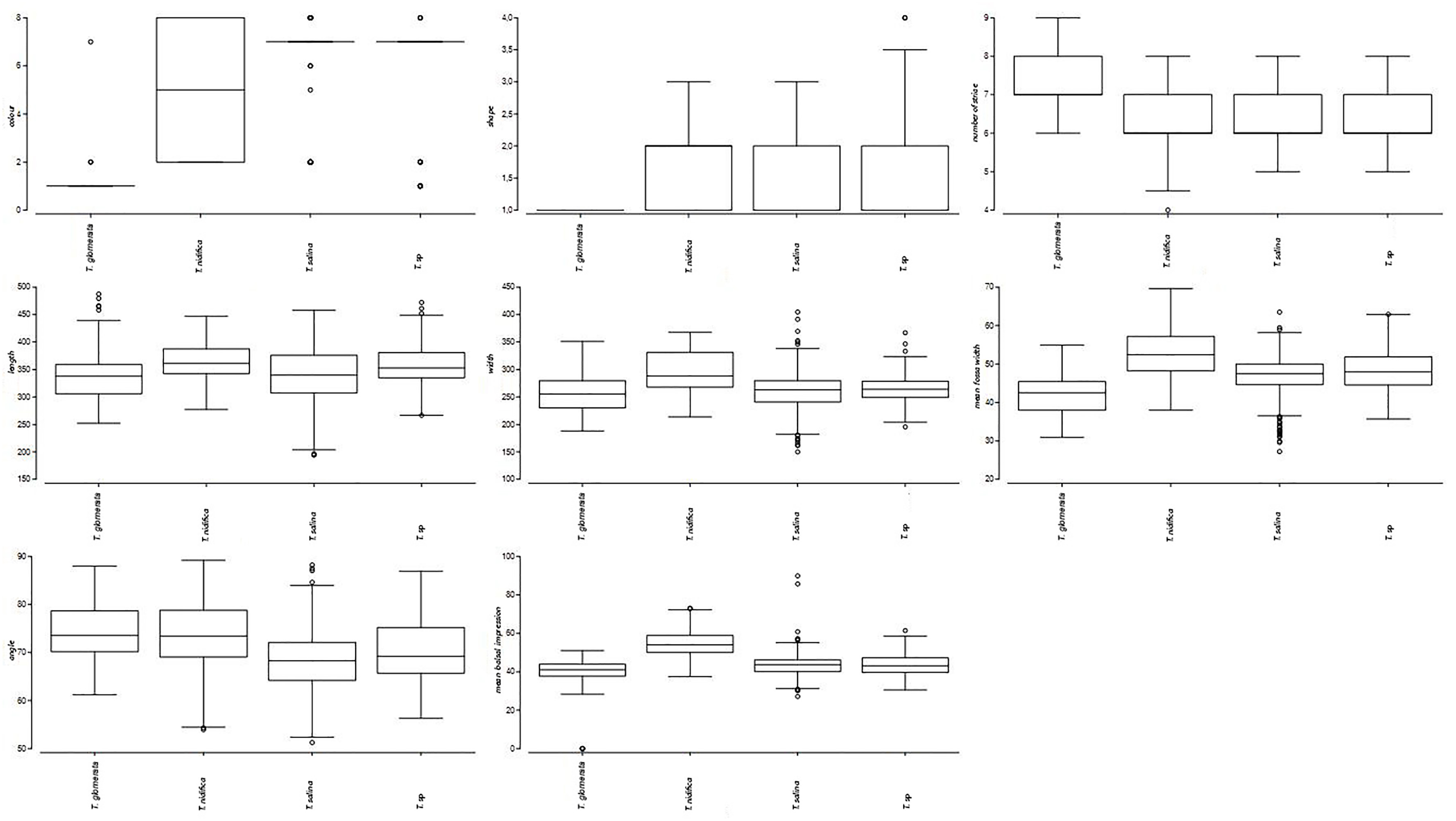

Supplement: Supplementary Figure 4 — Multiplot Analysis of T. glomerata, T. nidifica, T. salina and T. sp. Qualitative parameter are measured in µm. [file Image_4.jpeg]
